# Human Cytomegalovirus US28 Genomic Variation Among Infants at a Tertiary Care Hospital From Eastern India

**DOI:** 10.7759/cureus.91516

**Published:** 2025-09-03

**Authors:** Subham Ravi Nayak, Swaranjika Sahoo, Subham Kumar Padhi, Gaurav Verma, Swetapadma Jena, Santosh Kumar Panda, Manas Kumar Nayak, SKS Parashar, Dipti Pattnaik, Bidyut Kumar Das, A. Raj Kumar Patro

**Affiliations:** 1 Department of Microbiology, Kalinga Institute of Medical Sciences, Bhubaneswar, IND; 2 Department of Pediatrics, Kalinga Institute of Medical Sciences, Bhubaneswar, IND; 3 Department of Neonatology, Kalinga Institute of Medical Sciences, Bhubaneswar, IND; 4 Department of Paediatrics, Kalinga Institute of Medical Sciences, Bhubaneswar, IND; 5 Department of Nanosensor Laboratory, School of Applied Sciences, Kalinga Institute of Medical Sciences, Bhubaneswar, IND; 6 Department of Clinical Immunology and Rheumatology, S.C.B. Medical College and Hospital, Cuttack, IND

**Keywords:** chemokine receptor, genotyping, human cytomegalovirus (hcmv), infant, pcr, us28

## Abstract

Human cytomegalovirus (HCMV) poses a significant global health threat, particularly to newborns and immunocompromised individuals. HCMV exhibits extensive genetic diversity, and a specific genotype may be associated with virulence. HCMV US28 encodes for a chemokine receptor that plays a role in cellular processes and in pathogenesis. In this study, we investigated the HCMV US28 genotype distribution in infants.

Material and methods: 72 suspected infants during the study period were included in the study. Deoxyribonucleic acid (DNA) was extracted from clinical specimens using the spin column method, and the US28 ORF target gene was amplified using the US28-specific nested polymerase chain reaction (PCR). The housekeeping β-globin gene was amplified in each run. Amplified US28 gene products were PCR purified, and genotypes were discriminated by Sanger sequencing. Selected sequences were submitted to the National Center for Biotechnology Information (NCBI), USA, and accession numbers were obtained.

Results: Of the 72 infants examined, 21 were amplified for the US28 region using nested PCR. The sequencing revealed that the majority of the US28 fall in the A2 genotype in 80% and 20% in the A1 group. Single-nucleotide polymorphisms (SNPs) are seen in the sequences. Our findings show no significant association of a specific genotype with symptomatic HCMV infection.

Conclusions: This study reports that the US28 A2 genotype is prevalent in this region. This study is of value in understanding the epidemiology of circulating strains in this region. Further studies are required from different geographical regions with larger sample sizes to get the true picture of the findings.

## Introduction

Human cytomegalovirus (HCMV) is a widespread virus, infecting most people across the world. The majority of HCMV infections are asymptomatic in immunocompetent individuals, while severe clinical complications can manifest in neonates with congenital infection and in immunocompromised individuals [1,2]. The occurrence of congenital HCMV infection shows variability across populations, with reported prevalence rates ranging from 0.19% to 6.1% among different populations across the world [3-5]. At birth, the majority of infants infected with HCMV show no symptoms. However, symptomatic neonates may present with a varied range of conditions, including hepatosplenomegaly, microcephaly, petechiae, chorioretinitis, abnormal cerebrospinal fluid index, or jaundice [6,7]. Besides this sudden onset of symptoms, late developmental disabilities such as intellectual retardation, sensorineural hearing loss (SNHL), and vision impairment are major concerns in HCMV infection [8].

HCMV is the largest member of the beta herpesvirus family, possesses a double-stranded DNA genome of 230 kb in length, and encodes more than 167 gene products open reading frames (ORF), contributing to its complex biological behavior and interaction with the host [9]. HCMV displays significant genetic variability, with particular genotypes potentially linked to differences in virulence [10]. G protein-coupled receptors are a prominent group of transmembrane proteins that play a crucial role in regulating various cellular functions and hence are a druggable target [11]. The US28 open reading frame (ORF) encodes for a viral glycoprotein that functions as a homolog of cellular G protein-coupled receptors (GPCRs). This viral protein acts as a broad-spectrum β-chemokine receptor, effectively binding and interfering with the function of a diverse array of extracellular chemokines. This interference mechanism allows US28 to modulate host immune responses, primarily by altering chemokine-mediated immune cell recruitment and signaling [12,13].

HCMV-encoded G protein-coupled receptor, US28, expressed both lytic and latent phases of infection [14]. Genetic variability in this gene can increase the risk of severe clinical outcomes. Limited studies have examined HCMV genetic variability in the US28 region and its association with the variable disease outcomes [6,15,16]. In India, there is a scarcity of studies focusing on the HCMV US28 genetic variability, highlighting the necessity to identify the circulating strains in this region. This study aimed to investigate the HCMV US28 genotype distribution in HCMV-infected infants.

## Materials and methods

Study design and sample collection

This cross-sectional study enrolled infants admitted to the Pediatrics department at Kalinga Institute of Medical Sciences, Kalinga Institute of Industrial Technology, deemed to be a university, Bhubaneswar, from January 2024 to June 2024, a tertiary care hospital in the eastern region of India. Urine samples were collected from eligible infants with suspected HCMV infection. Infants were categorized as symptomatic if they exhibited clinical signs such as pallor, microcephaly, neonatal hepatitis, hydrocephalus, splenomegaly, or chorioretinitis, or if laboratory findings that raised suspicion included elevated transaminase levels, low platelets, or a positive CMV IgM test. Asymptomatic infants had no signs of disease but were included if either mother or infant had a positive CMV IgM test. Infants with immunodeficiency conditions, previous antiviral therapy, or genetic hearing loss were excluded [17]. The study was approved by the Institutional Ethics Committee of Kalinga Institute of Medical Sciences, KIIT University, Bhubaneswar (Ref No. KIIT/KIMS/IEC/1345/2023).

Nucleic acid extraction and HCMV US28 PCR

Nucleic acid extraction from the urine sample was carried out by a spin column-based extraction procedure using the QIAamp DNA Mini Kit (Cat: 51304, Qiagen, Germany) following the manufacturer's instructions. The extracted DNA samples were further subjected to PCR amplification by using primers from the N terminus of ORF of US28 [US28-Fw 5′-GTGAACCGCTCATATAGACC; US28-Rev 5′-GAAACAGGCAGTGAGTAACG-3′; and US28 heminested-Rev 5′-CATCCACAGAGGTAGTGTAC-3′], amplifying 429 bp and 366 bp [18] with cycling conditions of 94°C for 4 minutes, followed by 35 cycles of 94°C for 0:30 minutes, 55°C for 0:30 minutes, and 72°C for 0:45 minutes. This was followed by a single extension cycle of 72°C for 10 minutes. The known positive and known negative samples were included in each PCR run. The housekeeping b-globin gene was included for the adequacy of the sample in each run. The amplified PCR product was run in 2% agarose stained with ethidium bromide and visualized with a gel documentation system (Vilber, Germany).

Sequencing

The resulting PCR product was gel purified, and the purified amplicon was processed for Sanger sequencing using the Di-Deoxy chain termination method (BigDye™ Terminator v3.1 Cycle Sequencing) using the 3500 Genetic Analyzer (Applied Biosystems, USA) to deduce the sequence of the purified DNA [18,19]. The nucleotide sequences were aligned with published sequences from GenBank to assign a genotype using BioEdit Sequence Alignment Editor software [20,30], and phylogenetic trees were constructed using MEGA X [21,31]. The following reference sequences of US 28 genotypes were accessed from GenBank: genotypes A1, A2, A3, B1, B2, C, and D, with NCBI GenBank accession numbers KY490069, GU937742, AF498083, AF498084, OK000909, JX512198, KT726955, AF498085, KU550089, JX512204, KX544839, KT726949, and KT726946.

Statistical analysis

The median and interquartile range or number (frequency) are used to express data. GraphPad Prism software version 8.0.0 for Windows, GraphPad Software, San Diego, California, USA, was used for statistical analysis [32]. Fisher's two-tailed exact test was used for the analysis of variables. p<0.05 was chosen as the level of statistical significance.

## Results

**Figure 1:**
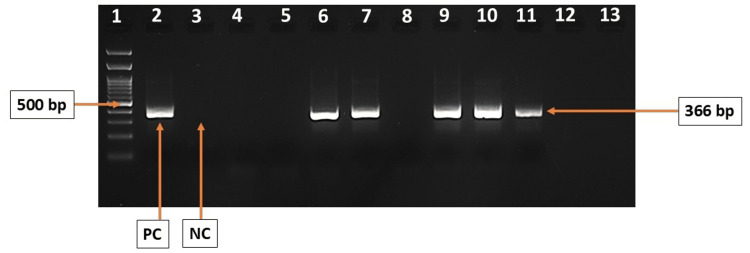
2% gel image showing HCMV US28 nested PCR amplicon of representative samples PCR: Polymerase chain reaction HCMV US28 nested PCR amplification (amplicon size 366 bp) Lane 1: 100 bp molecular weight marker (highlighted 500 bp), Lane 2: PC (positive control), Lane 3: NC (negative control), Lane 4-5, Lane 8, and Lane 12-13: samples negative for HCMV US28; Lane 6-7 and 9-11: samples positive for HCMV US28 gene. The image is created by the author.

During the study period, 72 infants were enrolled. Of these, 29% (21/72) tested positive for HCMV by US28 PCR (Figure 1). The median age of the infants was one month (ranging from 0.2 to 5.0 months). The median birth weight for the infants was 1330 grams with a range of 580 to 3100 grams. The median duration of hospital stay for the cohort was 1.00 months, with a range of 0.30 to 3.00 months.

**Figure 2:**
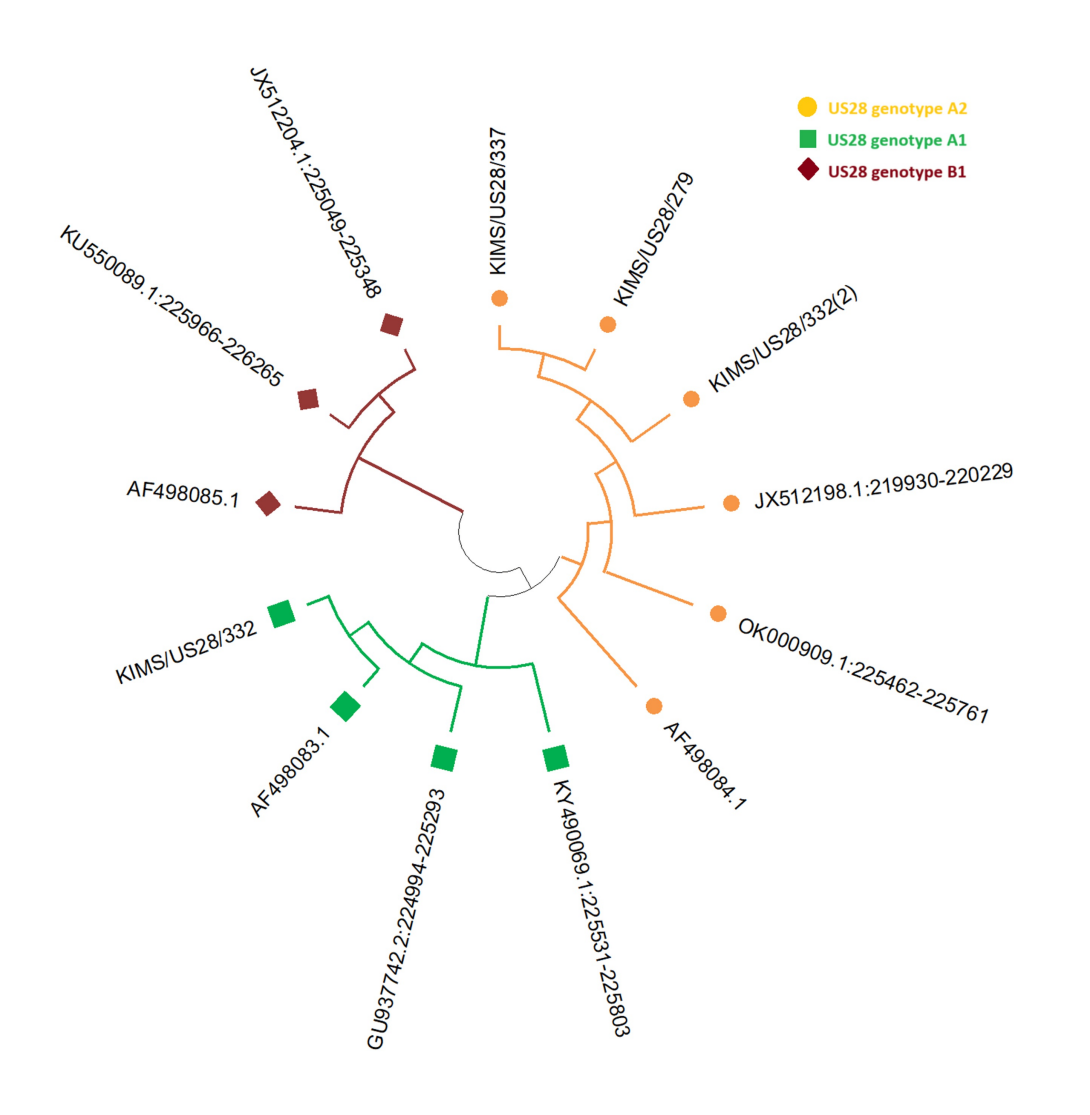
Phylogenetic analysis of US28 genotypes matched with the prototype sequences The image is created by the author.

**Figure 3:**
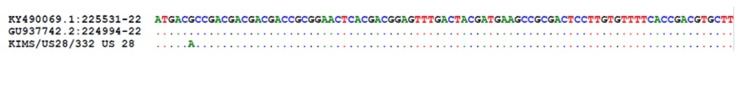
HCMV US28 type A1 aligned sequence of representative samples with reference genotype The image is created by the author.

**Figure 4:**
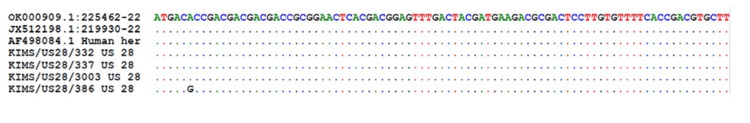
HCMV US28 type A2 aligned sequence of representative samples with reference genotype The image is created by the author.

Of the 21 HCMV-positive infants, 15 samples underwent genotyping using Sanger sequencing. Genotyping results of US28 reveal that 12 samples have genotype A2, whereas three samples have genotype A1 (Figure 2). Among the US28 A1 genotype, 2 were from symptomatic infants and 1 was from an asymptomatic infant. For the US28 A2 genotype, nine were from symptomatic infants and three were from asymptomatic infants. Among clinical findings, thrombocytopenia was seen in two cases in the US28 A1 genotype, whereas six cases were seen in the US28 A2 genotype (p=1.000); microcephaly was seen in one case in the US28 A1 genotype and 2 cases in the US28 A2 genotype (p=0.5165); hepatosplenomegaly was seen in one case in US28 A1, whereas six cases were seen in the US28 A2 genotype (p=1.000); and one cholestasis case was seen in US28 A1 vs. 5 cases seen in the US28 A2 genotype (p=0.5692). There was no difference in the genotype distribution among HCMV symptomatic and asymptomatic individuals (p=1.000) (Table 1). Representative sequences were submitted to NCBI GenBank, National Institutes of Health (NIH), USA, with Accession Nos. PV805648, PV805649, and PV805652 obtained. The alignment of US28 A2 with the prototype sequence reveals the following synonymous mutation in the region 112 A à G, while in US28 A1 type synonymous mutations are seen in the region 6 G à A (Figures 3, 4).

**Table 1:** HCMV US28 genotype in symptomatic and asymptomatic infants HCMV: Human cytomegalovirus

HCMV US28 Type (n=15)	Symptomatic	Asymptomatic	p-value
US28 A1 (n_1=_3)	2	1	1.0000
US28 A2 (n_2=_12)	9	3	

## Discussion

HCMV exhibits extensive genetic diversity [22,23]. HCMV ORFs code for various homologs of immunomodulator receptors such as US28. As a homolog to G protein-coupled receptor (GPCR), this viral protein plays an important role in immune evasion and also contributes to modulating the host cellular pathways [24]. Examining HCMV US28 polymorphism has a significant impact, as these genetic variations may play an important role in immune evasion, as antibody responses generated against one strain may not effectively neutralize a different strain [9]. The HCMV-encoded US28 G protein-coupled receptor induces caspase-dependent apoptosis [25]. A recent report demonstrated that the US28 genetic variability is associated with antibody levels and interaction with human chemokines [26,27]. This study highlights the distribution of the HCMV US28 genotype in infants.

In this study, the analysis of human cytomegalovirus (HCMV) US28 genotypes revealed a predominance of genotype A2, with an occurrence rate of 80% (12/15), while genotype A1 was found in 20% (3/15) of genotyped samples. These findings are consistent with studies across the globe. A study by Arav-Boger R et al. similarly reported a high prevalence, with genotype A accounting for 78% of HCMV-positive clinical isolates. Our results are also consistent with the findings of Paradowska E, et al. in Poland, which specifically demonstrated the predominance of the US28 A2 genotype among clinical isolates [15,28]. This correlation suggests a potential global or regional dominance of the A2 genotype. Further, it is supported by a study by Lee et al. (2019) on HCMV-infected mothers and infants, which also identified US28 genotype A2 as most prevalent [16]. Moreover, our finding of no significant difference in US28 genotype distribution between symptomatic and asymptomatic congenital infections is consistent with a study by Pati S.K. et al., which similarly found no correlation between the A genotype and symptomatic disease [18]. This lack of direct association suggests that while specific US28 genotypes may be more common, they may not be related to the clinical presentation. Alternatively, disease severity is likely a result of a complex interplay between multiple viral genes, viral load, and host-specific factors, such as the infant's immune status [4]. Although in this study the occurrence of other US28 genotypes like A3, B1, B2, C, D & E was not found, as observed by studies reporting other groups [15,28,29], suggesting that this particular viral strain may be more common in our geographic region. This study has a limitation by its sample size; however, the information regarding the genotype distribution will be valuable for understanding the epidemiology and developing intervention strategies. Future studies with larger cohorts are needed to confirm this association.

## Conclusions

In conclusion, our study found that the US28 genotype A2 was the most common strain of HCMV in our cohort of infected infants seen in this region. This study is of value in understanding the epidemiology of circulating strains in this region. Furthermore, studies are needed from different geographical regions with larger sample sizes to obtain a more accurate picture of the findings.
